# The conceptualisation and measurement of social frailty in older people: an umbrella review

**DOI:** 10.1016/j.tjfa.2025.100078

**Published:** 2025-08-05

**Authors:** Martin Webber, Beth Casey, Laura Tucker, Kirsty Shires, Mark Wilberforce, Barbara Hanratty, Louise Tomkow, David Sinclair, Jennifer Liddle, Dawn Sissons, Lynette Joubert

**Affiliations:** a Mental Health Social Care Research Centre, School for Business and Society, University of York, UK; b Population Health Sciences Institute, Faculty of Medical Sciences, Newcastle University, UK; c Division of Population Health, Health Services Research & Primary Care, School of Health Sciences, University of Manchester, UK; d Kingston upon Hull City Council, UK; e Department of Social Work, Melbourne School of Health Sciences, University of Melbourne, Australia

**Keywords:** Social frailty, Umbrella review, Systematic review, Social environment, Conceptualisation, Measurement

## Abstract

**Background:**

The social domain of frailty is often poorly defined or missing from measures of frailty. The literature is still inconclusive on the nature and scope of social frailty, although studies indicate that it has a substantial impact on disability and mortality outcomes.

**Aims:**

This Umbrella Review aimed to synthesise concepts and measures of social frailty.

**Methods:**

A search for reviews was performed in Web of Science, CINAHL, SOCINDEX, Medline, PsychoINFO and COSMIN databases. This Umbrella Review was conducted and reported with reference to the Joanna Briggs Institute (JBI) Reviewer’s Manual. The JBI Critical Appraisal Checklist for Systematic Review was used to assess the quality of studies.

**Results:**

Sixteen reviews were included. The concept of social frailty was summarised as weakness in a person’s social infrastructure compounded by a declining ability to change their circumstances. Forty-two measures of social frailty were identified which included a total of 228 items relating to social frailty. These were grouped into nine domains, of which participation in social activities was most commonly included within measures.

**Conclusion:**

The use of diverse indicators creates a challenge for the measurement of social frailty. Their limited use in health and social care practice undermines the practical utility of the concept. This review helps to provide conceptual clarity and a platform for the development and validation of a robust social frailty measure which will facilitate the identification of people at risk and target interventions to prevent or alleviate the impact of social frailty on older people.

## 1 Background

Frailty in older people is a health priority in an increasingly ageing world population. The concept of ‘frailty’ describes a distinctive, age-related state in which multiple body systems lose their in-built reserves [1]. It is predicated on physiological decline characterised by exhaustion, slowness, weight loss, low physical activity, and weakness [2], which limits adaptation to stressors [3]. Frailty is increasingly common with age [4], and a key influence on the need for social care, including 24 hour support [5]. Frailty is also conceptualised as the accumulation of deficits with increasing age [6], some of which may be social. Hitherto less recognised, social frailty may both augment and lead to physical disease processes, reduce quality of life and result in a downward spiral with increasing dependency and recurrent hospitalisation [7]. Where components of social frailty are compromised an older person’s ability to participate in the management of their long-term health conditions and their health outcomes are likely to be worse [8].

The concept of frailty, its measurement and interventions, has developed through a medical lens, though it is associated with adverse social factors, particularly reduced social networks and higher rates of loneliness [3]. While frailty is inherently a multidimensional concept, the social domain is often missing or captured by subjective or ambiguous questions [9]. Social frailty is a term that was introduced to encapsulate the notion of resources required to fulfil basic social needs and to more fully capture the social dimension of frailty. It was defined by Bunt et al. [10] as “a continuum of being at risk of losing, or having lost, social and general resources, activities, or abilities that are important for fulfilling one or more basic social needs during the life span” (p. 326). Living alone, having a reduced social network, experiencing a lack of social support, being lonely, and participating infrequently in social activities are factors that have been identified as determinants of social frailty [11].

Meta-analyses have found a pooled prevalence of social frailty of 18.8 % in community settings, although it varies from 4.9 % in China to 29.2 % in European urban centres [12]. It is predictive of certain adverse outcomes, such as disability, depression, reduced neuropsychological function and early mortality [[13], [14], [15]]. In addition, there is substantial evidence that both low received and perceived social support leads to worse outcomes in cerebral and cardiovascular health, such as incident coronary artery disease, incident congestive heart disease mortality, incident and recurrent stroke, and dementia and cognitive impairment [16,17]. However, the literature is still inconclusive on the nature and scope of social frailty and reveals a significant variety of approaches to the concept. It is less explored than other domains of frailty and presents higher complexity [11].

There is potential for overlap or conceptual confusion with the adjacent concept of social vulnerability, which has been operationalised and measured as an accumulation of social deficits [18]. Social vulnerability refers to cumulative factors which make people more susceptible to harm, such as poverty, limited access to resources or inadequate housing, for example. As such, it captures how external social structures influence health risks. In contrast, however, social frailty refers to a person’s declining ability to maintain social roles and relationships, and is more individual and functional in nature.

In order to target social interventions most effectively, practitioners working in health and social care services need clarity about the measurement of social frailty. Frailty, loneliness, and social isolation are all associated with adverse outcomes in older adults [19]. However, most studies investigating social factors influencing transition into residential care focus on a single or a limited number of determinants. Less is known about how adverse social factors can inform predictions of institutional care, despite these being more amenable to prevention or reversal than other relevant factors around cognitive decline and deteriorating physical health. There is a need to understand the concept of social frailty, and develop a way of measuring it that is both attuned to the needs of health and social care practitioners and sensitive to the needs and personal preferences of older people, to inform effective practice in this area.

The objective for this Umbrella Review is to map how social frailty is conceptualised and measured. This review aims to synthesise concepts and measures of social frailty. The review aims to work towards developing a set of transdisciplinary questions which measure social frailty that are practice-relevant and feasible for routine use by diverse health and social care practitioners.

The questions addressed by this review are:

1. How is social frailty conceptualised?
2. How are the social domains of frailty measured in frailty research?

## 2 Methods

An Umbrella Review methodology was used to synthesise knowledge from existing systematic reviews of social frailty. This Umbrella Review was conducted and reported with reference to the Joanna Briggs Institute (JBI) Reviewer's Manual [20]. A protocol for this review was developed but was not published, as this review was conducted within a brief development project which did not permit time for publication.

### 2.1 Inclusion criteria

*Participants:* People aged 60 and over

*Phenomena of interest:* Conceptualisation and/or measurement of social frailty or the social domains of frailty measures

*Type of studies:* Systematic reviews of any type published in a peer-reviewed journal in the English language since 2010

*Context:* People living in community settings in any international setting

### 2.2 Exclusion criteria

*Participants:* People with specific health conditions where specific frailty measures have been developed which are bespoke to that population

*Phenomena of interest:* Frailty research that does not include a social domain or where only a single item measuring social phenomena has been included; reviews which do not consider the conceptualisation or measurement of social frailty

*Type of studies:* Narrative literature reviews without a systematic methodology

*Context:* Inpatient or institutional populations

### 2.3 Search strategy

A pragmatic approach to searching was used to accommodate the short space of time available for the review to be conducted. Firstly, to identify reviews of the concept and measurement of social frailty, the following databases were searched: Web of Science, CINAHL, SOCINDEX, Medline and PsycINFO. These databases were selected as they provide a good coverage of international health and social care literature with minimal overlap. Variants of this search string were used in each database:

(“social frailty” OR “socially frail” OR “social frail*”) AND (“older people” OR “old*” OR “elder*” OR “gerontol*” OR “aging” OR “age*” OR “senior*” OR “geriatric*” OR “old adult” OR “old people” OR “older adults” OR “aged”) AND (“review*” OR “evidence synthesis”) (in title)

If the database had an option to filter a search for reviews, the latter search line was not included in the search string but the filter for reviews was applied instead. In addition, a limit to publications from 2010 onwards was placed on the search to ensure the review was contemporaneous.

Secondly, a search of the COSMIN database of systematic reviews of outcome measurement instruments using the search term “frailty” was conducted. This was an efficient way to identify reviews of frailty measures as this database uses robust search filters and criteria for inclusion.

### 2.4 Study screening and selection

The first stage of screening titles and abstracts was conducted by two reviewers independently and any disagreements were resolved through discussion. The full text of shortlisted abstracts were also independently reviewed by two reviewers. Any disagreements about eligibility were resolved through discussion. Study screening and selection was conducted in Covidence [21].

**Fig. 1:**
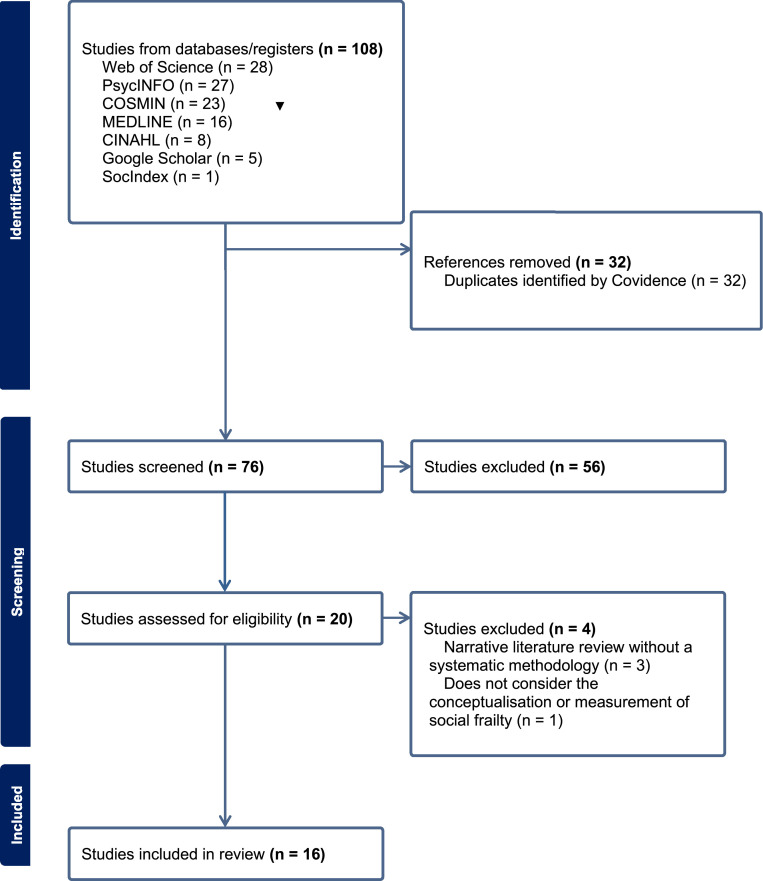
PRISMA flow diagram.

The PRISMA flow diagram for the review is depicted in Fig. 1. 76 papers were considered potentially eligible. Following title and abstract screening, 20 papers were selected for full text review with 16 papers meeting the inclusion criteria for the review.

### 2.5 Assessment of methodological quality

Each included review was assessed using the JBI Critical Appraisal Checklist for systematic reviews and research syntheses [22]. An overall appraisal score was generated by the tool which assisted decision-making about the inclusion of reviews in the review. Only reviews which scored highly were included in the review, though none were excluded.

### 2.6 Data extraction

The following data was extracted from the 16 review papers to answer our research questions:

• Author(s) and date
• Aims/purpose
• Method
• Study population
• Inclusion criteria
• Definitions or conceptualisations of social frailty identified
• Social frailty measures or frailty measures that include a social component

One reviewer extracted the information and a second reviewer checked the accuracy of the extracted information in a random sample of one-third of papers (*n* = 6).

From the measures identified in the reviews, one reviewer extracted all the questions which related to social frailty. To ensure all questions were included, the reviewer checked the original measure as well as the papers included in the reviews. 42 measures were identified and a second reviewer checked the extracted questions from a random sample of one-third of measures (*n* = 15).

### 2.7 Data summary

Extracted data were summarised in two tables. Table 1 below presents the characteristics of the reviews. Two reviewers studied the definitions and conceptualisations of social frailty extracted from the reviews and summarised common elements. A new definition and conceptualisation was discussed and amended iteratively with advisory groups of practitioners and people with lived experience, and agreed by the research team, which reflected the scholarship on social frailty.

**Table 1:** Definitions and measures of social frailty in included reviews.

**Author, date**	**Review aims**	**Method**	**Study population**	**Inclusion criteria**	**Definitions / conceptualisations of social frailty identified**	**Measures of social frailty**	**JBI appraisal score**
Bautista & Malhotra (2018)	To identify and measure frailty in Singapore to glean insights that can inform the selection of valid measurement tools for use in research and clinical settings.	Scoping review	People aged 60 years or over	Reported empirical research based on primary and/or secondary data; conducted in Singapore; specified the identification, measurement and/or assessment of frailty; and used a clear definition of frailty	Summarised common definitions of frailty within the papers reviewed. Social frailty: "a multifaceted concept that involves a continuum of being at risk of losing, or having lost general or social resources, social behaviours and activities, and self-management abilities that are important for fulfilling basic social needs"	Tilburg Frailty Index; Social Frailty Index	11
Bessa et al. (2018)	To review frailty assessment instruments (screening tools and severity measures) with a focus on their social components.	Systematic review	People aged 60 years or over	Reference to frailty as the main term; studies published in English; studies that describe and test the operationalization of multidimensional assessment tool specifically developed for the assessment and identification of frailty; studies including the assessment of frailty by at least one social variable/question.	Social components of frailty vary from instrument to instrument and cover the concepts of social isolation, loneliness, social network, social support and social participation.	Social components in the following tools: Postal Screening Questionnaire (PSQ); Frailty Staging System (FSS); Sherbrooke Postal Questionnaire (SPQ); Frailty Index (FI); Groningen Frailty Indicator (GFI); Vulnerable Elders Survey-13 (VES-13); Frailty Index – Comprehensive Geriatric Assessment (FI - CGA); Edmonton Frail Scale (EFS); Prisma-7; Tilburg Frailty Indicator (TFI); Comprehensive Frailty Assessment Instrument (CFAI); Social Frailty Phenotype (SFP); Easycare Two-Step Older Persons Screening (Easycare-TOS); Gérontopôle Frailty Screening Tool (GFST); Comprehensive Model of Frailty (CMF); Kihon Checklist (KCL); Questionnaire to define Social Frailty Status (QSFS); Frailty Groupe Iso-Ressource Evaluation FRAGIRE; interRAI Home Care Frailty Scale iRHCFS; Social Frailty Index (SFI)	8
Bunt et al. (2017)	To evaluate existing insights on social frailty, and structure and synthesize these insights in a scoping review, using the social needs concept of SPF theory as heuristic for ordering and structuring these insights.	Scoping review	Older adults (no ages were specified)	Papers included if they described the concept of social frailty, contained a definition or determinants of social frailty, or social determinants of overall frailty, or if they contained a combination of all of these criteria	Social frailty can be understood as a multidimensional concept - social resources (or restrictions), social behaviors and activities, and self-management abilities (which all impact on affecting social needs). Social frailty can be defined as a continuum of being at risk of losing, or having lost, social and general resources, activities, or abilities that are important for fulfilling one or more basic social needs during the life span.	N/A	9
Dent et al. (2016)	To determine which operationalisations of frailty were best at measuring frailty according to Clegg's guidelines of frailty classification: which measurements could accurately identify frailty; which could reliably predict patient outcomes and response to potential therapies; and which were based on biological theory.	Scoping review	People aged 65 years or over	Published between 2009 and 2015; frailty objectively measured in either observational, cross-sectional or randomised controlled trials; English language	N/A	Table of 29 frailty measures was included though only these included social dimensions: Tilburg Frailty Indicator (TFI); PRISMA-7; Groningen Frailty Indicator (GFI); Edmonton Frailty Score (EFS).	8
de Vries et al. (2011)	To assess frailty instruments on clinimetric properties and to search for the best available frailty instrument that can be used as an evaluative outcome measure in clinical practice and that is useful in observational and experimental studies.	Systematic review	‘Frail elderly' used as a search term but no age limit specifed	Published before February 2010; an instrument was interpreted being a frailty instrument when the authors explicitly defined that the instrument intends to measure the level of frailty; studies explicitly and operationally describe a measurement instrument	The following definition was used as a starting point: ‘Frailty is a dynamic state affecting an individual who experiences losses in one or more domains of human functioning (physical, psychological, and social), which is caused by the influence of a range of variables and which increases the risk of adverse outcomes’ (Gobbens et al., 2010a, p. 85). Risk factors that are mentioned (based on recent reviews) in relation to social dimensions of frailty - in the social dimension: lack of social contacts and social support (Markle-Reid and Browne, 2003; Gobbens et al., 2007).	Frailty measures included in the review that include social relations/social support: Frailty Index; Groningen Frailty Indicator (GFI); Clinical Global Impression of Change in Physical Frailty (CGIC-PF); Instrument ‘Winograd’; Geriatric Functional Evaluation (GFE); Frailty Index Comprehensive Geriatric Assessment (FI-CGA)	9
Durepos et al. (2022)	To map the breadth of primary studies; and describe the meaning, perceptions, and perceived implications of frailty language amongst community-dwelling older adults.	Scoping review	Older adults living in the community	Published in English between 1994 and 2019; studies describe the perceptions, meaning and perceived implications of frailty language, and/or the diagnosis of frailty	Participants described social frailty as losses/decline in social interactions, feelings of loneliness (Puts et al., 2009), and disengagement behaviours (e.g., refusing invitations to social gatherings, reducing phone calls to peers/family members) (Age UK, British Geriatrics Society, 2015; Warmoth, Lang, et al., 2016), which compounded losses that were common in later life. Individuals living with social frailty were described as withdrawing from participation in social events, while at the same time being excluded or not invited to activities because of limitations (Warmoth, Lang, et al., 2016). Social isolation was therefore perceived as a cause and result of frailty. Being excluded from social activities was perceived as reducing motivation to participate in future events, which led to further isolation (Warmoth, Lang, et al., 2016). Participants explained that social frailty was exacerbated by environmental constraints (e.g., limited access to transportation, poor building accessibility, financial concerns) that resulted in social disconnection (Age UK, British Geriatrics Society, 2015; Escourrou et al., 2017, 2019).	Fit-Frail Scale (Theou et al., 2019)	8
Hamid et al. (2024)	To characterize the social frailty domains and their health outcomes by reviewing the frailty screening tools in older people living in the Asia-Pacific region	Systematic review	Community-dwelling adults aged 60 years or over	Studies published between 2002 and 2023 in English; must include empirical evidence published in peer-reviewed journals; Asia-Pacific region; must include the assessment of the social domain of frailty.	Social frailty could describe a lack of frequent participation in social events, networks, and contact and insufficient support, leading to serious health outcomes. Social frailty can be defined as the loss of one or more human social resources essential for fulfilling basic human needs throughout life (Bunt et al., 2017). The conceptual bases of this study were developed from Gobbens’s and Bunt’s frameworks on the determinants of social frailty (Bunt et al., 2017; Gobbens et al., 2010). Gobbens et al. (2010) suggested three main frailty domains: physical, psychological, and social. Gobbens et al. (2010) highlighted two sub-domains for social frailty, including social network and social support; achieving these domains enhances the quality of life and well-being as people age. Bunt et al. (2017) confirmed that social frailty in old age is affected by four social factors: social needs, social resources (such as social supports and social networks), social behaviors or social activities, and general resources (indirect way of fulfilling social needs, such as living situation, educational level, and income or financial status).	Kaigo-Yobo Checklist (KYCL); Social Frailty Questionnaire (SFQ); Kihon Checklist (KCL); The Chinese version of the Lubben Social Network Scale (LSNS-6); Social Frailty Questionnaire (SFQ); Social Frailty Screening Questionnaire (HALFT); Social Deficits/Social Frailty Questionnaire (SD-SF); Social Vulnerability Index (SVI); Social Frailty Questionnaire (SFQ); Tilburg Frailty Indicator (TFI); Chinese Comprehensive Frailty Assessment Instrument (CFAI); Frailty Assessment Scale (FAS); Chinese version of Comprehensive Geriatric Assessment-Frailty Index (CGA-FI); Comprehensive Model of Frailty (CMF)	11
Huang et al. (2021)	To describe the existing research on frailty measurement of older people and to understand their characteristics, with a focus on conceptual definitions, psychometric properties, and diagnostic accuracies.	Narrative review	People aged 65 years or over	Studies published between 2001 and 2020; study aimed to develop a quantitative frailty measurement; the measurement is preferred with frailty classification or frailty prognosis as a kind of outcome prediction.	The starting point of an integral conceptual definition of frailty is a dynamic state affecting an individual who experiences losses in one or more domains of human functioning (ie, physical, psychological, and social), which is caused by the influence of a range of variables and which increases the risk of adverse outcomes. Gobbens et al. defined frailty with human functioning losses of one or more domains of “Physical,” “Psychological” and “Social.” Social relations and support, poverty, low education, area deprivation, and living alone may be grouped into social domains.	Measures described as 'multi-dimensional' or including a social domain - Frailty Index derived from Comprehensive Geriatric Assessment (FI-CGA); Groningen Frailty Indicator (GFI); Comprehensive Frailty Assessment Instrument (CFAI);Edmonton Frailty Scale (EFS); Frailty Index of Accumulative Deficits (FI-CD); Geriatric Functional Evaluation (GFE); Gérontopôle Frailty Screening Tool (GFST); Kihon Checklist (KCL); PRISMA-7 Questionnaire; Sherbrooke Postal Questionnaire (SPQ); Tilburg Frailty Indicator (TFI); Winograd's Instrument	9
Jia (2024)	To ascertain the prevalence rates and risk factors of social frailty in older adults	Systematic review and meta-analysis	People aged 55 years or over living in the community	Studies included a measure of social frailty; prevalence data on social frailty among older adults; quantitative studies including case-control, cross sectional and cohort studies.	Social frailty can be defined as the continuous risk of losing or having lost social and general resources, activities or abilities that are important for the satisfaction of one or more basic social needs over a lifetime (Bunt et al., 2017)	HALFT measure of social frailty; Makizako’s social frailty index (MSFI); Bunt's social frailty concept; social frailty phenotype; Tilburg Frailty Indicator (TFI); Japanese version of the Lubben Social Network Scale (LSNS-6); social frailty screening index (SFSI); comprehensive frailty assessment instrument (CFAI)	10
Khalil & Gobbens (2023)	To address frailty attributes, risk factors, consequences, perceptions, and lived experiences of older adults with frailty.	Descriptive review	People aged 60 years or over	Papers in English which included frailty as the main concept, including a definition; frailty risk factors; frailty consequences; frailty perceptions; and lived experience perspectives.	Social frailty is a continuum from being at risk of losing to having lost resources that are important for fulfilling one or more basic social needs during the life span (Gobbens et al., 2010). These resources, per this definition, can be social resources such as children or spouses, social behaviours and activities such as social participation, and self-management abilities such as feeling empowered and autonomous in making decisions.	The Mixed Conceptual Model of Frailty described risk factors; perception and lived experiences of older adults and consequences for society, healthcare, family and caregivers.	10
Lee et al. (2023)	To explore how researchers applied the Tilburg Frailty Indicator (TFI) to older adults living in all possible settings and which pathway of the Integral Conceptual Model of Frailty was tested using the TFI in older adults living in all possible settings.	Scoping review	Older people (no ages were specified)	Studies related to the use of the ICMF or TFI; written in English; published in full text articles; and study designs were longitudinal.	Social frailty is a continuum from being at risk of losing to having lost resources that are important for fulfilling one or more basic social needs during the life span (Gobbens et al., 2010)	Tilburg Frailty Indicator (TFI) and Integrated Conceptual Model of Frailty (ICMF)	10
Rasiah et al. (2022)	To understand how instruments were developed to assess frailty in community dwelling older adults	Systematic review	People aged 65 years or over	Discussed instruments used to measure frailty (including instrument development) in community dwelling older adults 65 years of age or older; instruments developed for research purposes or in clinical practice or both; and published in English.	Multidimensional perspective of frailty including physical, cognitive, psychological, socioeconomic, nutritional and social; deficit approach; housing and interaction of social support, demographic and biopsychosocial factors; clinically and socially constructed; vulnerability. Other definitions of frailty can be grouped as either a state or syndrome, as influenced by the two dominant measures of frailty in research and clinical practice - Frailty Index (Mitnitski et al., 2001) and Fried Phenotype (Fried et al., 2001)	4-Item Social Frailty Index; 7- Item Social Frailty Questionnaire; Tilburg Frailty Indicator	11
Sezgin et al. (2019)	To identify and examine definitions of frailty using qualitative thematic analysis	Systematic review and qualitative meta-aggregative review	Older people (no ages were specified)	Studies with any design that incorporated a definition of frailty or discussed its characteristics; conducted in any setting including primary care, secondary care, general practice or residential care settings; peer-reviewed; published between 2000 and 2018, in English with available full texts.	Multidimensional construct of frailty included social domain. Social networks and environment were cited as associated factors of frailty	N/A	10
Sezgin et al. (2020)	To identify and examine definitions of pre-frailty in the literature to characterise important features and factors contributing to the construct	Systematic review and qualitative meta-aggregative review	Older people (no ages were specified)	Studies reporting a definition of pre-frailty conducted in any setting including residential care, general practice and secondary care; published in English between 2000 and 2018 with available full texts.	Defined social pre-frailty as a component of pre-frailty, which was characterised by no or insufficient social support or carer network. Social pre-frailty was considered present if people displayed one of the following: they went out less frequently compared with last year, did not visit friends, did not feel they were of use to friends or family, lived alone, or did not talk with someone every day.	Fried Frailty Phenotype (CHS); Frailty Index (FI); FRAIL Scale; Tilburg Frailty Indicator and Comprehensive Geriatric Assessment	10
Yu (2023)	To explore the prevalence of social frailty among older adults; and to identify the associated factors such as countries, age, research sites, and years that affect the levels of social frailty in older adults	Systematic review and meta-analysis	People aged 60 years or over	Studies reported the prevalence of social frailty in older adults; measures of social frailty are explicitly mentioned; quantitative observational study design (cohort, case-control, and cross-sectional study); published in English or Chinese.	Social frailty is defined as the lack of social resources, social activities, and self-management abilities that are necessary for meeting fundamental social demands. Some studies have found that demographic and sociological factors such as education level, marital status, financial burden, residence, and monthly income can deeply impact social frailty.	N/A	11
Zhang (2023)	To conduct a meta-analysis synthesizing the pooled prevalence of social frailty among older adults, and to identify which factors could influence the prevalence of social frailty among older adults.	Systematic review and meta-analysis	People aged 60 years or over	Studies that reported the prevalence of social frailty, using a clear definition of social frailty; cross-sectional and cohort studies, regardless of language or country.	Based on the concept of social needs in the social production functions theory, social frailty was defined as a continuum of being at risk of losing, or having lost social resources, social behaviours, social activities, and self-management abilities to fulfil basic social needs.	Makizako Social Frailty Index; Social Frailty Screening Index; Tilburg Frailty Indicator; HALFT scale; LSNS-6; Accumulated functional deficits	11

Table 2 comprised a matrix of measures (presented in rows) and domains of social frailty (presented in columns). Questions were inserted in the matrix according to the concept they were measuring. The selection of domains was informed by an initial grouping of the questions into related concepts. This was reviewed by the research team and amended iteratively. For example, the domain ‘social networks’ was separated into two concepts: ‘social isolation’, which included questions on the number and frequency of social contacts, and ‘social capital’, which incorporated questions about the perceived *quality* of these social contacts and relationships, including the benefits and resources gained from these relationships and interactions. The final iteration of the table included 9 domains: social isolation; social capital (defined as resources accessible to people through their networks); formal social support (professional or formal support); social activities (frequency of participation and factors impacting on ability to attend); loneliness; living alone; work (including voluntary work); social role (the ability to help others and feelings of helpfulness towards friends or family); and life events. An additional domain of ‘socio-demographic and neighbourhood context’ was used to include questions on finances, age, marital status, education, housing and neighbourhoods as these measured contextual components associated with social frailty.

**Table 2:** Domains, questions and response options in social frailty measures.

**Domain** **Measure**	**Social activities**	**Social capital**	**Social isolation**	**Living alone**	**Loneliness**	**Social role**	**Life events**	**Work**	**Formal social support**	**Socio-demographic context**
**Accumulated functional deficits** [45]		- People without help *(yes; no)* - Lack of help in daily activities in the past 3 months *(yes; no)*	- Not having regular contact with family *(yes; no)* - Not having regular contact with friends or neighbours *(yes; no)*	- Living alone *(yes; no)*						
**Bunt’s social frailty concept** [10]	- Participation in social activities *(regular=0; none=1)*		- Total scores of the Lubben Social Network Scale *(<12=1; ≥12=0)*	- Living alone *(yes=1; no=0)*						- Need financial support *(yes=1; no=0)*
**Chinese Comprehensive Frailty Assessment Instrument (CFAI)** [46]		- I have more than enough people to rely on when I'm in trouble - I know a lot of people I can fully trust - I have enough people close to me *(strongly disagree; disagree; neutral; agree; strongly agree)*			- I feel empty - I want someone to be around - I often feel excluded *(strongly disagree; disagree; neutral; agree; strongly agree)*					- The house is in poor condition or poorly preserved - The house is not very comfortable - Heating a house is difficult - I don't feel comfortable enough in my room - I don't like the neighbourhood around *(strongly disagree; disagree; neutral; agree; strongly agree)*
**Chinese version of Comprehensive Geriatric Assessment-Frailty Index (CGA-FI)** [47]									- Uses formal home supports? (yes; no)	
**Clinical Global Impression of Change in Physical Frailty (CGIC-PF)** [37]			- Interaction with others *(clinician makes notes in column)*			-Roles *(clinician makes notes in column)*	-Life events *(clinician makes notes in column)*			-Living situation *(clinician makes notes in column)*
**Comprehensive Frailty Assessment Instrument (CFAI)** [48]		- Suppose you are unable to carry out the activities you usually do in the house-keeping for a certain while, whom would you be able to appeal to? *(relation to person(s) is noted)* - I know many people whom I can totally trust - There are enough people with whom I feel a bond - There are plenty of people whom I can rely on when I am in trouble. *(completely disagree; disagree; neither agree nor disagree; agree; completely agree)*			- I experience a general sense of emptiness - I miss having people around me - I often feel rejected *(completely disagree; disagree; neither agree nor disagree; agree; completely agree)*					Which statements are applicable to your home? - House is in a bad condition poorly kept - House is not very comfortable - It is difficult to heat the house - Insufficient comfort in the house - I do not like the neighbourhood *(not applicable at all; rather not applicable; neither applicable nor inapplicable; rather applicable; completely applicable)*
**Comprehensive Model of Frailty (CMF)** [49]	- Frequency of attending social activities *(weekly; more frequent)* - Presence of barriers to social activities *(strongly disagree; disagree; neutral; agree; strongly agree).*	- Having a spouse or a child to confide with when they need emotional support *(yes; no)*		- Living alone *(yes; no)*	- Often feels lonely *(yes; no)*					- Perception of one’s household being “worse-off” or “mediocre” compared with an average local household.
**Edmonton Frail Scale (EFS)** [50]		- When you need help, can you count on someone who is willing and able to meet your needs? *(always; sometimes; never)*								
**Evaluative Frailty Index for Physical Activity (EFIPA)** [51]	- Are there activities that someone else has taken over for you recently? - Are there enough organized activities for you nearby? - Do you have problems getting out for organized activities (e.g. problems with transportation to get to them)? *(most of the time; sometimes; rarely)*	- When you need help, are there people who are willing and able to help you? *(most of the time; sometimes; rarely)*			- Do you feel lonely? *(most of the time; sometimes; rarely)*				- Do you have enough help from professionals? *(most of the time; sometimes; rarely)*	- Do you have any housing problems? *(most of the time; sometimes; rarely).*
**Easycare Two-step Older persons Screening (first step)** [52]		- Social network *(sufficient and strong social network; large but weak social network; small but strong social network; small and weak or no social network)*			- Loneliness *(no loneliness; had complaints of loneliness in the past 12 months; unknown)*					
**Easycare Two-step Older persons Screening (second step)** [52]	- Are you able to pursue leisure, interests, hobbies, work and learning activities which are important to you? *(yes; no)* - How often in the past 4 weeks have your physical health or emotional problems hampered your social activities (such as visits to friends or close family members)? *(continuously; mostly; sometimes; rarely; never)*	- Is there anyone who would be able to help you in case of illness or emergency? *(yes; no)*	- Do you have contact with people in your neighbourhood? *(with few people / little contact; with few people / but sufficient contact; with many people / little contact; with enough people sufficient contact)*	- Do you live alone? *(yes; no)*	- Do you feel lonely? *(never; sometimes; often)*		- Have you suffered from any recent loss or bereave-ment? *(yes; no)*			- What is the highest level of education that you have completed? *(7 choices tick box*) - Marital status *(married; divorced; widow / widower / partner deceased; unmarried; long-term cohabitation / unmarried)* - In what kind of accomm-odation do you live? *(10 options tick box)*
**Frailty Assessment Scale (FAS)** [53]	- Do you participate in any of the following meetings or activities? (social; religious; cultural; leisure; civic or social groups; interest and political groups; volunteer groups; learning organizations) *(yes; no)*	- Is there someone available to you whom you can count on to listen to you when you need to talk? *(yes; no)*	- Do you have as much contact as you would like with someone you feel close to? *(yes; no)*							
**Frailty Staging System (FSS)** [54]		- Who will able to help you in case of illness or emergency? *(actual and potential caregivers must be identified)*								
**Frailty Index – Comprehensive Geriatric Assessment (FI - CGA)** [55]				- Living alone *(yes; no)*					- Uses formal home supports *(yes; no)*	- Institution-alised *(yes; no)*
**Frailty Groupe Iso-Ressource Evaluation (FRAGIRE)** [56]	- Do you participate in sport activities? - Do you use internet? *(not at all; a little; quite a bit; very much)*				- Have you felt lonely or abandon-ment? *(not at all; a little; quite a bit; very much)*	- Do you assist relative(s) you feel responsible for? *(not at all; a little; quite a bit; very much)*				- Your financial situation seemed sufficient to meet your needs? *(not at all; a little; quite a bit; very much)*
**Frailty Index (FI)** [57]	- Social activities limitations *(major limitation; minor limitation; no limitation)*				- Loneliness *(all the time; sometimes; never)*			-Work limitations *(major limitation; minor limitation; no limitation)*		
**Frailty Index (FI)** [58]	- Changes in social activities *(decline or no decline in participation in social activities in last 90 days)*		- Social isolation *(never or hardly ever alone, or for about one hour, during the day; alone for long periods of time or all the time*)		- Feels lonely *(does not feel lonely; feels lonely)*					
**Frailty Index** [59]	- Participation in social activities *(yes; occasionally; no).*		- Alone for long periods of time or all the time *(yes; occasionally; no).*					- Work performing *(yes; occasionally; no).* - Doing housework *(yes; occasionally; no).*		
**Frailty Index** [60]	- During the past 4 weeks, how much of the time has your physical health or emotional problems interfered with your social activities (like visiting friends, relatives, etc.)? *(all of the time; most of the time; some of the time; a little of the time; none of the time)* - During the past 4 weeks, to what extent has your physical health or emotional problems interfered with your normal social activities with family, friends, neighbours, or groups? *(extremely; quite a bit; moderately; slightly; not at all)*									
**Frailty Index** [61]			- Daily contact with other people through meetings, phone contacts, emails, etc. *(presence; absence)*		- Do you feel lonely? *(presence; absence)*					
**Frailty Index** [62]	- Problems with transport when you want to go out *(yes; sometimes; no)*	- There are people in my life that really care about me. - Feeling that in their neighbour-hood people generally trust each other *(yes; sometimes; no)*		- Lives alone *(yes; no)*						- Married *(yes; no)* - Family’s money situation *(bad; good)* - Feeling their neighbourhood as a safe place *(yes; sometimes; no)* - Feeling safe in your own home *(never; most of the time; some of the time; always)*
**Frailty Index for Japanese Elderly** [63]	- Less outdoor activity - Fewer hobbies or interests - Lower daily physical activity *(yes; no)*		- Less contact with neighbours - Less friendships other than neighbours *(yes; no)*							
**Gérontopôle Frailty Screening Tool (GFST)** [64]				- Does your patient live alone? *(yes; no; don’t know).*						
**Groningen Frailty Indicator (GFI)** [65]					- Do you sometimes experience emptiness around yourself? - Do you sometimes miss people around yourself? - Do you sometimes feel abandoned? *(yes; no)*					
**HALFT scale** [66]	- Limited social participation in the previous 12 months *(yes; no)*		- Not having anyone to talk to every day *(yes; no)*		- Loneliness in the past week *(yes; no)*	- Inability to help others within the past 12 months *(yes; no)*				- Financial difficultly over the past 12 months *(yes; no)*
**interRAI Home Care Frailty Scale iRHCFS** [67]	- Decline in social activities - Reduced social activities - Withdrawal from activities of interest *(yes; no)*									
**Kaigo-Yobo Checklist (KYCL)** [68]	- How often do you usually go out? *(more than once per 2–3 days or less than once a week)* - Do you usually stay at home all day long? - Do you have any hobbies? *(yes; no)*	- Do you have neighbours who you can talk closely with? *(yes; no)*	- Do you have close friends, family, or relatives who you visit? *(yes; no)*							
**Kihon Checklist (KCL)** [69]	- Do you go out at least once a week? - Do you go out less frequently compared to last year? *(yes; no)*	- Do you turn to your family or friends for advice? *(yes; no)*	- Do you sometimes visit your friends? *(yes; no)*							
**Lubben Social Network Scale (LSNS-6)** [70]		- How many relatives do you feel close to such that you could call on them for help? - How many relatives do you feel at ease with that you can talk about private matters? - How many friends do you feel close to such that you could call on them for help? - How many friends do you feel at ease with that you can talk about private matters? *(none; one; two; three or four; five-eight; nine or more)*	- How many relatives do you see or hear from at least once a month? - How many friends do you see or hear from at least once a month? *(none; one; two; three or four; five-eight; nine or more)*							
**Makizako Social Frailty Index** [71]	- Do you go out less frequently compared with last year? *(yes; no)*		- Do you sometimes visit your friends? - Do you have friends you talk to by telephone? - Do you talk with someone every day? *(yes; no)*	- Do you live alone? *(yes; no)*		- Do you feel you are helpful to friends or family? *(yes; no)*				
**Modified social frailty index** [72]	- Do you participate in any community activities or volunteer activities? *(yes; no)*		- Do you sometimes visit your friends? *(yes; no)*	- How many people do you live with? *(alone; with others)*						- Do you have a financial problem in your daily life? *(yes; no)*
**Postal Screening Questionnaire (PSQ)** [73]	- Are you confined to your home through ill health? *(yes; no)*	- Are you without a relative you could call on for help? *(yes; no)*		- Do you live on your own? *(yes; no)*						
**PRISMA-7** [74]	- In general, do you have any health problems that require you to limit your activities? - In general, do you have any health problems that require you to stay at home? *(yes; no)*	- In case of need, can you count on someone close to you? *(yes; no)*								- Are you older than 85 years? - Are you male? *(yes; no)*
**Sherbrooke Postal Questionnaire (SPQ)** [75]				- Do you live alone? *(yes; no)*						
**Social Deficits/Social Frailty Questionnaire (SD-SF)** [76]	- Social participation in 8 types of activities: social club, religious services, cultural, sports / leisure, civic / social, political / interest group, volunteer work, and learning / education group *(almost never; more frequently)*	- Emotional support: have someone who can listen to concerns or worries - Instru-mental support: have someone who can help with house-work, cooking,etc.) - Care support received from family members, relatives, friends or neighbours *(strongly disagree; disagree; neutral; agree; strongly agree).*	- The number of close relatives, friends, or neighbours *(none; one or more)* - Frequency of contact with close relatives, friends or neighbours *(rarely; more frequently).*	- Living alone *(yes; no)*		- Support provided to family members, relatives, friends. *(strongly disagree; disagree; neutral; agree; strongly agree).*				- Education *(none; at least primary)* - Household income *(in quartiles)* - Marital status *(married; not married)*
**Social Frailty Phenotype (SFP)** [77]		- Do you have family and/or friends you could ask for help if you needed assistance? - Is there anyone special that you can trust and talk to about personal matters and your feelings? - In the past 3 months, have you failed to receive help from others with shopping, food preparation, house cleaning, ironing or other personal activities even though you needed help? *(yes; no)*	- How often do you meet or talk to your closest relatives? *(less than once a week; every day; every 2 or 3 days; weekly; monthly; once a year)* - How often do you meet or talk to your friends and/or neighbours? *(less than once a week; every day; every 2 or 3 days; weekly; monthly; once a year)*	- Living alone *(yes; no)*						
**Social Frailty Screening Index** [78]	- How often in the last month did you participate in social activity? *(almost daily; almost every week; not regularly; none)*		- How often in the last month did you visit your friends? *(almost daily; almost every week; not regularly; none)*							
**Social Frailty Questionnaire** [79]	- Frequency of social activities across 6 activity categories *(rarely or not at all; more frequently)*	- Do you have someone to confide in? *(yes; no)* - Frequency of visits or calls with family, friends or loved ones (assessed via two different questions) *(none or no more than once a year; more frequently)* - Receives little help when required *(none to a very little; at least some)*		- Who do you live with? *(alone; with others)*						- Education *(none; at least primary)* - Are you limited by your financial resources to pay for needed medical service? *(to a great extent; other)* - Housing type: *(living in 1–2 room public housing; other)*
**Social Frailty Scale (SFS-8)** [80]	- Do you go out less frequently compared with last year? *(yes; no)*	- Do you turn to family or friends for advice? - Do you have someone to confide in? *(yes; no)*	- Do you sometimes visit your friends? - Do you talk with someone every day? - Do you eat with someone at least once a day? *(yes; no)*	- Do you live alone? *(yes; no)*						- Are you limited by your financial resources to pay for needed medical service? *(yes; no)*
**Social Frailty Screening Index** [81]	- How often do you participate in the following groups: volunteer, sports, hobby, learning or cultural, nursing care prevention, senior citizens, or residents’ associations? *(four or more times a week; two or three times a week; once a week; one to three times a month; a few times in a year; never)*	- How do you get along with your neighbours? *(I have a neighbour who comes and goes to each house; I have a neighbour to chat with on the street; I have a neighbour to say hello to; I do not communicate with neighbours)*		- Do you live alone? *(yes; no)*						- Are you satisfied with your economic condition? *(very satisfied; satisfied; unsatisfied, very unsatisfied)*
**Social Vulnerability Index (SVI)** [82]	- Do not participate in any groups *(yes; no)*	- Feel close to few people or relatives - Cannot find somebody to help with daily chore - Do not know somebody can turn to with personal issues - Do not trust at least one person’s advice - Could not find someone to care for house - Do not have somebody to talk about important decisions - Don’t have people to help with things like shopping etc. *(yes; no)*	- See relatives once a month - No close friends - I rarely meet/talk with family or friends - Hearing cause difficulty when visiting friends - Unable to see to recognize friend across a street *(yes; no)*	- Living alone *(yes; no)*	- When lonely, there is no one to talk to *(yes; no)*	- People do not talk to you about important decisions *(yes; no)*		- Do not do regular volunteer work *(yes; no)*		- Present marital status - Low yearly household income *(yes; no)*
**Tilburg Frailty Indicator (TFI)** [83]		- Do you receive enough support from other people? *(yes; no)*		- Do you live alone? *(yes; no)*	- Do you sometimes miss having people around you? *(yes; no)*		- Have you experien-ced one or more of the following events during the past year? - the death of a loved one - a serious illness yourself / loved one - a divorce or ending of an important intimate relationship - a traffic accident - a crime *(yes; no)*			- Which sex are you? - What is your age? - What is your marital status? - What is the highest level of education you have completed? - Which category indicates your net monthly household income? - Are you satisfied with your home living environment? *(yes; no)*
**Number of times domain features in measures**	26	23	20	18	14	6	3	3	3	19

## 3 Results

### 3.1 Summary of included reviews

Six reviews focused on measures and assessments of frailty [[23], [24], [25], [26], [27], [28]] and a further two specifically focused on the social dimensions of frailty assessments [9,29]. Three reviews focused on the conceptualisation and definition of frailty and social frailty [10,30,31]. Three systematic reviews and meta-analyses looked at the prevalence and associated factors of social frailty [12,32,33]. The final two reviews explored older people’s perceptions and experiences of frailty and social frailty [34,35]. The reviews were predominantly international in scope, though Bautista and Malhotra [23] only included papers from Singapore and Hamid et al. [29] focused only on the Asia-Pacific region.

### 3.2 Summary of conceptualisation of social frailty

The definition of social frailty arising from the summary of conceptualisations in the reviews (Table 1) is: *weakness in a person’s social infrastructure compounded by a declining ability to change their circumstances*. An individual’s ‘social infrastructure’ refers to the people they are in contact with who add value to their lives. Weaknesses in a person’s social infrastructure may be experienced as a lack of family or friends, limited interactions with other people or an absence of social support, for example. It is also visible in reduced social participation or an inability to meet basic social needs. People may also experience loneliness or social isolation. These weaknesses in a person’s social infrastructure are compounded by having a declining ability to address contextual factors such as problems with housing; having limited financial resources; or experiencing access problems with local transport or within local communities.

Papers discussed the multi-dimensional nature of frailty and many referred to Gobbens et al.’s [36] conceptual model of frailty, which includes a social dimension, as a key framework [12,24,26,28,29,35]. Other reviews reported on the complexity of defining and conceptualising frailty, including its dimensions or sub-domains (including, for example, physical, social, socio-economic, cognitive and psychological); whether or not it should be deficit focused, a continuum or a transitional state; and its associations with adverse health outcomes [30,31].

In terms of social frailty, Bunt et al.’s [10] definition was widely cited within reviews [12,23,29,32,33]. Otherwise, reviews referred to it as an absence, decline or loss of social networks, social relations and interactions [26,29,34] and an absence of or insufficient social support [26,27,29]. Moreover, in Durepos et al.’s [34] review, older adults described social frailty in relation to feelings of loneliness and ‘disengagement behaviours’ such as declining invitations to social events. Social isolation was also perceived as a cause and consequence of frailty. Exclusion from social activities was viewed as reducing motivation to participate in the future, leading to further social isolation.

**Fig. 2:**
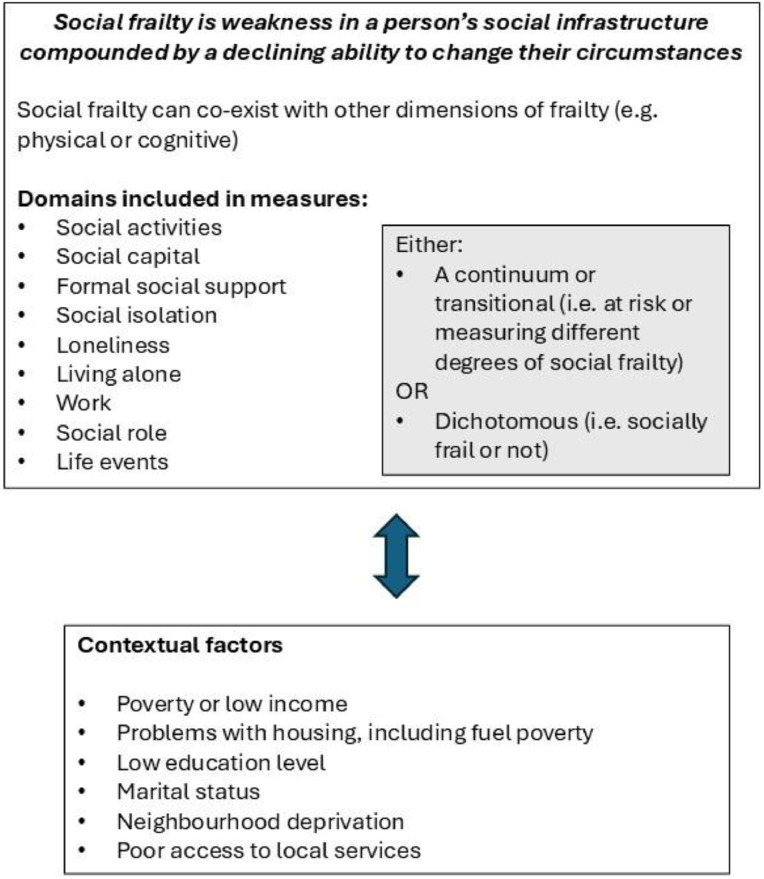
Conceptual framework of social frailty.

In addition to findings on conceptualising social frailty, several papers highlighted associated risks and influencing factors. Socio-demographic factors included: living in poverty, financial constraints and low monthly income [25,26,33]; area deprivation; living alone [25,26]; low education level [25,26,33]; housing and residence [27,33] and marital status [33]. Further, two papers [33,34] highlighted wider environmental factors, including limited access to transport and building accessibility, which can lead to social disconnection. Fig. 2 summarises the findings of the review and presents a conceptual framework of social frailty. This includes the definition arising from the summary of conceptualisations from Table 1 and the measurement domains from Table 2.

### 3.3 Summary of measures of social frailty

Three reviews explored questions included in measures of social frailty. Bessa et al. [9] found that the social components of frailty measures differed between instruments and included the dimensions of social isolation, loneliness, social network, social support and social participation. Bessa et al. [9] noted that the instruments included in their review placed less emphasis on the social dimension of frailty compared to physical and psychological domains. Similarly, de Vries et al. [24] reported that only five instruments in their review considered all three domains of frailty (physical, psychological and social), with the majority only considering physical frailty. Furthermore, out of 32 frailty instruments in Rasiah et al.’s [27] study, social frailty was included in only three measures.

Our review identified 42 measures of social frailty which included a total of 228 items (Table 2). Of the nine domains included in Table 2, ‘social activities’ was most frequently used with 26 measures including questions on this domain. These predominantly asked about going out, participating in social activities or barriers preventing people from doing so. The second most frequent domain was ‘social capital’ (defined here as resources accessible to people through their social networks), which was included within 23 measures. Commonly cited social resources accessible through networks assessed by these questions included emotional support, practical support and neighbourhood connections. Twenty included questions on social isolation and 18 measures asked whether the person lives alone. Loneliness was also a common domain and included within 14 measures. The other domains were included in fewer measures (social role (*n* = 6); life events (*n* = 3); work (*n* = 3); and formal social support (*n* = 3)).

Nineteen measures included socio-demographic questions, typically relating to finances, age, marital status, education and housing. These were included in Table 2 when they were cited as core components of the measures, though it is recognised that these data are often routinely collected in research and clinical practice but may not have been included within other measures. In our definition above, these contextual factors compound a person’s social infrastructure, highlighting the importance of social context to the measurement of social frailty, though are not included as an additional domain of social frailty.

The measures included between 1 and 15 questions relating to social frailty, although none included questions in all the domains. The response options were predominantly dichotomous (e.g. ‘do you live alone?’ (*yes/no*)); ordinal (e.g. ‘how often in the last month did you visit your friends?’ (*1. Almost daily 2. Almost every week 3. Not regularly 4. None*)); or nominal (e.g. ‘how many friends do you see or hear from at least once a month?’ (*0 = none, 1 = one, 2 = two, 3 (three/four), 4 (five-eight) and 5 (nine/more*)). Just one measure asked open questions: the Clinical Global Impression of Change in Physical Frailty [37] was based on clinician judgement and prompted them to make notes on, for example, ‘interaction with others’. In general, the approach to determining cut-off points for social frailty appeared rather arbitrary, with limited psychometric analysis undertaken.

## 4 Discussion

The literature on social frailty is large and growing. This Umbrella Review of reviews provides a summary of the conceptualisation and measurement of social frailty. We found that there is a lack of consensus around how to define social frailty. However, existing literature appears to define social frailty as *weakness in a person’s social infrastructure compounded by a declining ability to change their circumstances*. An individual’s agency to effect change typically does not feature in existing measures, which are mostly descriptive of an individual’s social situation. In fact, the large variety of domains included in the measures indicate a lack of agreement about the dimensions of social frailty. The use of diverse indicators creates a challenge for the measurement of social frailty and potentially undermines the integrity of the concept.

The derivation of measures appears to have been driven more by pragmatism than clinical utility. They are predominantly derived from social surveys or longitudinal panel studies where questions are defined by social scientists rather than clinicians – or indeed older adults living with social frailty. The measures are often post-hoc composites of social indicators, typically weighting them equally, despite the likelihood that different dimensions have varying relationships with, and predictive power for, frailty. For example, there is evidence that poor social support, loneliness and social isolation each predict future physical frailty [38,39], but they are separate constructs and the respective contribution of each needs to be considered. It is also likely that the dimensions are subjectively experienced differently by older people, with some more important than others [40].

This review found that the most frequent domain used in social frailty measures is participation in social activities. This is possibly because it is more tangible and easier to measure than subjective indicators such as loneliness. In addition, the indicators of physical frailty such as exhaustion, slowness, low physical activity and weakness may make it more difficult for people to go out and participate in social activities. Conversely, not taking part in social activities may be an early indicator of frailty, as it is for depression [41]. Conceptually, there is a link between functional ability and social activity, though in the design of social frailty measures it is important not to make assumptions that people want to go out. It is possible that some people prefer to connect with others via telephone or online. Also, reduced mobility does not necessarily equate to frailty. A robust social environment can mitigate risks associated with reduced mobility or health problems in older age.

Loneliness was also a common domain of social frailty measures, possibly reflecting concern about its high prevalence among older adults in many countries [42]. Its interaction with other social frailty domains is likely to be complex, as it may be both a domain and an outcome. For example, loneliness could be a consequence of a decrease in the quality and quantity of social relationships, making it an outcome of social frailty. It could also arise from physical frailty (which itself could pose barriers to interventions which address loneliness) [43]. However, as a subjective domain of social frailty, it could help to distinguish between those who feel distressed by their social environment and those who do not. One study found a bidirectional association between loneliness and social frailty, indicating that it may be considered an integral component of social frailty rather than an outcome of it [44]. More research is required to explore the complex relationship between loneliness and social frailty, as it could be modelled as a predictor, component, mediator or outcome of social frailty.

A multi-dimensional assessment of social frailty is required to fully understand an older person’s social environment and accurately detect risks for frailty. This measure needs to be subject to rigorous psychometric evaluation in clinical settings so that it can be incorporated into routine assessment of frailty by clinicians [15]. Robust social frailty measures will facilitate the identification of people at-risk and will enable the targeting of social interventions to address areas of need. Some social risk factors for frailty may be preventable or could be addressed through targeted help or support. Similar conclusions have been drawn from the allied literature on social vulnerability, where it has been argued that social interventions can help to prevent frailty [5]. The lack of routine use of reliable social frailty measures in health and social care services means that opportunities for early and effective intervention are potentially being missed.

We conducted a rigorous search of six databases to identify high quality systematic reviews. Whilst we cannot guarantee this was exhaustive, the reviews provided comprehensive coverage of the social frailty literature. However, a limitation of this Umbrella Review is that the social frailty measures were identified within reviews rather than through a specific search for them. It is possible that some were not included, though any omissions are likely to be minimal. A further limitation is that the review only included English language publications, so may have omitted some reviews. Also, this review did not undertake a formal thematic analysis of definitions of social frailty, though the process of summarising them and reaching a consensus statement was rigorous. This involved iterative reviews by advisory groups of practitioners and people with lived experience, and the research team.

## 5 Conclusion

This review has summarised a substantial literature on social frailty through its review of 16 systematic reviews. It has reached a consensus definition of the concept and highlighted the diversity of domains used in measures. It found a lack of consistency in the measurement of social frailty, which may undermine the usefulness of the concept. It also highlighted the need to develop a measure which is valid and reliable for use in clinical practice so that the concept could take on some practical utility. Having a clear definition and an overview of existing measurement strategies will provide a firm foundation for the development and validation of a clinically-relevant measure.

## Funding

This project was funded by the 10.13039/100010377National Institute for Health and Care Research (NIHR) Three Research Schools Prevention Research Programme [Grant Reference Number NIHR20400 – Prev]. The views expressed are those of the authors and not necessarily those of the NIHR or the Department of Health and Social Care.

## Declaration of generative AI and AI-assisted technologies in the writing process

We have not used AI in the writing of this manuscript.

## CRediT authorship contribution statement

**Martin Webber:** Writing – review & editing, Writing – original draft, Supervision, Project administration, Methodology, Investigation, Funding acquisition, Formal analysis, Data curation, Conceptualization. **Beth Casey:** Writing – review & editing, Writing – original draft, Project administration, Investigation, Formal analysis, Data curation. **Laura Tucker:** Writing – review & editing, Project administration, Methodology, Investigation, Formal analysis, Data curation. **Kirsty Shires:** Writing – review & editing, Formal analysis. **Mark Wilberforce:** Writing – review & editing, Methodology, Funding acquisition, Formal analysis, Conceptualization. **Barbara Hanratty:** Writing – review & editing, Funding acquisition, Formal analysis, Conceptualization. **Louise Tomkow:** Writing – review & editing, Funding acquisition, Formal analysis, Conceptualization. **David Sinclair:** Writing – review & editing, Funding acquisition, Formal analysis, Conceptualization. **Jennifer Liddle:** Writing – review & editing, Funding acquisition, Formal analysis, Conceptualization. **Dawn Sissons:** Writing – review & editing, Formal analysis. **Lynette Joubert:** Writing – review & editing, Writing – original draft, Project administration, Methodology, Investigation, Funding acquisition, Formal analysis, Data curation, Conceptualization.

## Declaration of competing interest

The authors declare the following financial interests/personal relationships which may be considered as potential competing interests: Martin Webber reports financial support was provided by National Institute for Health and Care Research. If there are other authors, they declare that they have no known competing financial interests or personal relationships that could have appeared to influence the work reported in this paper.

## References

[1] Clegg A., Young J., Iliffe S., Rikkert M.O., Rockwood K. (2013). Frailty in elderly people. Lancet.

[2] Fried L.P., Tangen C.M., Walston J., Newman A.B., Hirsch C., Gottdiener J. (2001). Frailty in older adults: evidence for a phenotype. J Gerontol.

[3] Hoogendijk E.O., Smit A.P., van Dam C., Schuster N.A., de Breij S., Holwerda T.J. (2020). Frailty combined with loneliness or social isolation: an elevated risk for mortality in later life. J Am Geriatr Soc.

[4] Sinclair D.R., Maharani A., Chandola T., Bower P., Hanratty B., Nazroo J. (2022). Frailty among older adults and its distribution in England. J Frailty Aging.

[5] Hanlon P., Wightman H., Politis M., Kirkpatrick S., Jones C., Andrew M.K. (2024). The relationship between frailty and social vulnerability: a systematic review. Lancet Healthy Longev.

[6] Rockwood K., Mitnitski A. (2011). Frailty defined by deficit accumulation and geriatric medicine defined by frailty. Clin Geriatr Med.

[7] Kojima G., Liljas A.E.M., Iliffe S. (2019). Frailty syndrome: implications and challenges for health care policy. Risk Manag Heal Policy.

[8] Reynolds R., Dennis S., Hasan I., Slewa J., Chen W., Tian D. (2018). A systematic review of chronic disease management interventions in primary care. BMC Fam Pr.

[9] Bessa B., Ribeiro O., Coelho T. (2018). Assessing the social dimension of frailty in old age: a systematic review. Arch Gerontol Geriatr.

[10] Bunt S., Steverink N., Olthof J., van der Schans C.P., Hobbelen J.S.M. (2017). Social frailty in older adults: a scoping review. Eur J Ageing.

[11] Bessa B., Coelho T., Ribeiro Ó. (2021). Social frailty dimensions and frailty models over time. Arch Gerontol Geriatr.

[12] Zhang X.-M., Cao S., Gao M., Xiao S., Xie X., Wu X. (2023). The prevalence of social frailty among older adults: a systematic review and meta-analysis. J Am Med Dir Assoc.

[13] Li X., Gao L., Qiu Y., Zhong T., Zheng L., Liu W. (2023). Social frailty as a predictor of adverse outcomes among older adults: a systematic review and meta-analysis. Aging Clin Exp Res.

[14] Ragusa F.S., Veronese N., Smith L., Koyanagi A., Dominguez L.J., Barbagallo M. (2022). Social frailty increases the risk of all-cause mortality: a longitudinal analysis of the english longitudinal study of ageing. Exp Gerontol.

[15] Goto T., Kishimoto T., Fujiwara S., Shirayama Y., Ichikawa T. (2024). Social frailty as a predictor of all-cause mortality and functional disability: a systematic review and meta-analysis. Sci Rep.

[16] Castro MdL, M Alves, Papoila A.L., Botelho A., Fragata J. (2023). One-year survival after cardiac surgery in frail older people - social support matters: a prospective cohort study. J Clin Med.

[17] Valtorta N.K., Kanaan M., Gilbody S., Ronzi S., Hanratty B. (2016). Loneliness and social isolation as risk factors for coronary heart disease and stroke: systematic review and meta-analysis of longitudinal observational studies. Heart.

[18] Andrew M.K., Mitnitski A.B., Rockwood K. (2008). Social vulnerability, frailty and mortality in elderly people. PLoS One.

[19] Hanratty B., Stow D., Collingridge Moore D., Valtorta N.K., Matthews F. (2018). Loneliness as a risk factor for care home admission in the english longitudinal study of ageing. Age Ageing.

[20] Aromataris E., Fernandez R., Godfrey C., Holly C., Khalil H., Tungpunkom P., Aromataris E., Lockwood C., Porritt K., Pilla B., Jordan Z. (2024). JBI manual for evidence synthesis.

[21] Veritas Health Innovation. Covidence systematic review software. Melbourne, Australia2025.

[22] Aromataris E., Fernandez R., Godfrey C.M., Holly C., Khalil H., Tungpunkom P. (2015). Summarizing systematic reviews: methodological development, conduct and reporting of an umbrella review approach. Int J Evid Based Healthc.

[23] Bautista M.A.C., Malhotra R. (2018). Identification and measurement of frailty: a scoping review of published research from Singapore. Ann Acad Med Singap.

[24] de Vries N.M., Staal J.B., van Ravensberg C.D., Hobbelen J.S.M., Olde Rikkert M.G.M., Nijhuis-van der Sanden M.W.G (2011). Outcome instruments to measure frailty: a systematic review. Ageing Res Rev.

[25] Dent E., Kowal P., Hoogendijk E.O. (2016). Frailty measurement in research and clinical practice: a review. Eur J Intern Med.

[26] Huang EY-z, Lam S.C. (2021). Review of frailty measurement of older people: evaluation of the conceptualization, included domains, psychometric properties, and applicability. Aging Med.

[27] Rasiah J., Gruneir A., Oelke N.D., Estabrooks C., Holroyd-Leduc J., Cummings G.G. (2022). Instruments to assess frailty in community dwelling older adults: a systematic review. Int J Nurs Stud.

[28] Lee J., Kim G.S., Kim S., Park J., Lee H., Shim M.-S. (2023). Use of the Tilburg frailty indicator in longitudinal studies with older adults: a scoping review. J Adv Nurs.

[29] Hamid T.A., Salih S.A., Zillah Abdullah S.F., Ibrahim R., Mahmud A. (2024). Characterization of social frailty domains and related adverse health outcomes in the Asia-Pacific: a systematic literature review. PeerJ.

[30] Sezgin D., O'Donovan M., Cornally N., Liew A., O'Caoimh R. (2019). Defining frailty for healthcare practice and research: a qualitative systematic review with thematic analysis. Int J Nurs Stud.

[31] Sezgin D., Liew A., O'Donovan M.R., O'Caoimh R. (2020). Pre-frailty as a multi-dimensional construct: a systematic review of definitions in the scientific literature. Geriatr Nurs.

[32] Jia B., Wang Z., Zhang T., Yue X., Zhang S. (2024). Prevalence of social frailty and risk factors among community-dwelling older adults: a systematic review and meta-analysis. Arch Gerontol Geriatr.

[33] Yu S., Wang J., Zeng L., Yang P., Tang P., Su S. (2023). The prevalence of social frailty among older adults: a systematic review and meta-analysis. Geriatr Nurs.

[34] Durepos P., Sakamoto M., Alsbury K., Hewston P., Borges J., Takaoka A. (2022). Older adults’ perceptions of frailty language: a scoping review. Can J Aging /Rev Can Du Vieil.

[35] Khalil A.H., Gobbens R.J.J. (2023). What if the clinical and older adults’ perspectives about frailty converge? A call for a mixed conceptual model of frailty: a traditional literature review. Healthcare.

[36] Gobbens R.J.J., Luijkx K.G., Wijnen-Sponselee M.T., Schols J.M.G.A. (2010). Towards an integral conceptual model of frailty. J Nutr Health Aging.

[37] Studenski S., Hayes R.P., Leibowitz R.Q., Bode R., Lavery L., Walston J. (2004). Clinical global impression of change in physical frailty: development of a measure based on clinical judgment. J Am Geriatr Soc.

[38] Ding Y.Y., Kuha J., Murphy M. (2017). Multidimensional predictors of physical frailty in older people: identifying how and for whom they exert their effects. Biogerontology.

[39] Davies K., Maharani A., Chandola T., Todd C., Pendleton N. (2021). The longitudinal relationship between loneliness, social isolation, and frailty in older adults in England: a prospective analysis. Lancet Healthy Longev.

[40] Cloutier-Fisher D., Kobayashi K., Smith A. (2011). The subjective dimension of social isolation: a qualitative investigation of older adults' experiences in small social support networks. J Aging Stud.

[41] Roh H.W., Hong C.H., Lee Y., Oh B.H., Lee K.S., Chang K.J. (2015). Participation in physical, social, and religious activity and risk of depression in the elderly: a community-based three-year longitudinal study in Korea. PLoS One.

[42] Chawla K., Kunonga T.P., Stow D., Barker R., Craig D., Hanratty B. (2021). Prevalence of loneliness amongst older people in high-income countries: a systematic review and meta-analysis. PLoS One.

[43] Mansfield L., Victor C., Meads C., Daykin N., Tomlinson A., Lane J. (2021). A conceptual review of loneliness in adults: qualitative evidence synthesis. Int J Env Res Public Health.

[44] Ye L., Bally E., Korenhof S.A., Fierloos I., Alhambra Borrás T., Clough G. (2024). The association between loneliness and frailty among community-dwelling older adults in five European countries: a longitudinal study. Age Ageing.

[45] Díaz-Alonso J., Bueno-Pérez A., Toraño-Ladero L., Caballero F.F., López-García E., Rodríguez-Artalejo F., Lana A. (2021). Limitación auditiva y fragilidad social en hombres y mujeres mayores. Gac Sanit.

[46] Qiao X., Wang C., Tian X., Dong L., Liu N., Jin Y., Si H. (2018). Cross-cultural adaptation and validation of the comprehensive frailty assessment instrument in Chinese community-dwelling older adults. Geriatr Gerontol Int.

[47] Ma L., Tang Z., Zhang L., Sun F., Li Y., Chan P. (2018). Prevalence of frailty and associated factors in the community-dwelling population of China. J Am Geriatr Soc.

[48] De Witte N., Gobbens R., De Donder L., Dury S., Buffel T., Schols J., Verté D. (2013). The comprehensive frailty assessment instrument: development, validity and reliability. Geriatr Nurs.

[49] Kwan J.S.K., Lau B.H.P., Cheung K.S.L. (2015). Toward a comprehensive model of frailty: an emerging concept from the Hong Kong centenarian study. J Am Med Dir Assoc.

[50] Rolfson D.B., Majumdar S.R., Tsuyuki R.T., Tahir A., Rockwood K. (2006). Validity and reliability of the Edmonton frail scale. Age Ageing.

[51] de Vries N.M., Staal J.B., Olde Rikkert M.G.M., Nijhuis-van der Sanden M.W.G (2013). Evaluative frailty index for physical activity (EFIP): a reliable and valid instrument to measure changes in level of frailty. Phys Ther.

[52] van Kempen J.A., Schers H.J., Jacobs A., Zuidema S.U., Ruikes F., Robben S.H. (2013). Development of an instrument for the identification of frail older people as a target population for integrated care. Br J Gen Pr.

[53] Kim K.J., Choi J., Shin J., Kim M., Won C.W. (2021). Consensus on components of frailty using the Delphi method: Korean frailty and aging cohort study. J Nutr Health Aging.

[54] Lachs M.S., Feinstein A.R., Cooney L.M., Drickamer M.A., Marottoli R.A., Pannill F.C., Tinetti M.E (1990). A simple procedure for general screening for functional disability in elderly patients. Ann Intern Med.

[55] Jones D., Song X., Mitnitski A., Rockwood K. (2005). Evaluation of a frailty index based on a comprehensive geriatric assessment in a population based study of elderly Canadians. Aging Clin Exp Res.

[56] Vernerey D., Anota A., Vandel P., Paget-Bailly S., Dion M., Bailly V. (2016). Development and validation of the FRAGIRE tool for assessment an older person’s risk for frailty. BMC Geriatr.

[57] Myers V., Drory Y., Goldbourt U., Gerber Y. (2014). Multilevel socioeconomic status and incidence of frailty post myocardial infarction. Int J Cardiol.

[58] McKenzie K., Ouellette-Kuntz H., Martin L. (2015). Using an accumulation of deficits approach to measure frailty in a population of home care users with intellectual and developmental disabilities: an analytical descriptive study. BMC Geriatr.

[59] Ma L., Zhang L., Tang Z., Sun F., Diao L., Wang J. (2016). Use of the frailty index in evaluating the prognosis of older people in Beijing: a cohort study with an 8-year follow-up. Arch Gerontol Geriatr.

[60] Young A.C., Glaser K., Spector T.D., Steves C.J. (2016). The identification of hereditary and environmental determinants of frailty in a cohort of UK twins. Twin Res Hum Genet.

[61] Bäckman K., Joas E., Falk H., Mitnitski A., Rockwood K., Skoog I. (2017). Changes in the lethality of frailty over 30 years: evidence from two cohorts of 70-year-olds in Gothenburg Sweden. J Gerontol A.

[62] Dent E., Dal Grande E., Price K., Taylor A.W (2017). Frailty and usage of health care systems: results from the South Australian monitoring and surveillance system (SAMSS). Maturitas.

[63] Yamanashi H., Shimizu Y., Nelson M., Koyamatsu J., Nagayoshi M., Kadota K. (2015). The association between living alone and frailty in a rural Japanese population: the Nagasaki islands study. J Prim Health Care.

[64] Tavassoli N., Guyonnet S., Abellan Van Kan G., Sourdet S., Krams T., Soto M.E. (2014). Description of 1108 older patients referred by their physician to the “Geriatric Frailty Clinic (G.F.C) for assessment of frailty and prevention of disability” at the gerontopole. J Nutr Health Aging.

[65] Bielderman A., van der Schans C.P., van Lieshout M.-R.J., de Greef M.H.G., Boersma F., Krijnen W.P., Steverink N. (2013). Multidimensional structure of the Groningen frailty indicator in community-dwelling older people. BMC Geriatr.

[66] Ma L., Sun F., Tang Z. (2018). Social frailty is associated with physical functioning, cognition, and depression, and predicts mortality. J Nutr Health Aging.

[67] Morris J.N., Howard E.P., Steel K.R. (2016). Development of the interRAI home care frailty scale. BMC Geriatr.

[68] Shinkai S., Yoshida H., Taniguchi Y., Murayama H., Nishi M., Amano H. (2016). Public health approach to preventing frailty in the community and its effect on healthy aging in Japan. Geriatr Gerontol Int.

[69] Arai H., Satake S. (2015). English translation of the Kihon checklist. Geriatr Gerontol Int.

[70] Lubben J., Blozik E., Gillmann G., Iliffe S., von Renteln Kruse W., Beck J.C., Stuck A.E. (2006). Performance of an abbreviated version of the Lubben social network scale among three European community-dwelling older adult populations. Gerontologist.

[71] Makizako H., Shimada H., Tsutsumimoto K., Lee S., Doi T., Nakakubo S. (2015). Social frailty in community-dwelling older adults as a risk factor for disability. J Am Med Dir Assoc.

[72] Nagai K., Tamaki K., Kusunoki H., Wada Y., Tsuji S., Itoh M. (2020). Physical frailty predicts the development of social frailty: a prospective cohort study. BMC Geriatr.

[73] Barber J.H., Wallis J.B., McKeating E. (1980). A postal screening questionnaire in preventive geriatric care. J R Coll Gen Pr.

[74] Raîche M., Hébert R., Dubois M.-F. (2008). PRISMA-7: a case-finding tool to identify older adults with moderate to severe disabilities. Arch Gerontol Geriatr.

[75] Hébert R., Bravo G., Korner-Bitensky N., Voyer L. (1996). Predictive validity of a postal questionnaire for screening community-dwelling elderly individuals at risk of functional decline. Age Ageing.

[76] Lee Y., Chon D., Kim J., Ki S., Yun J. (2020). The predictive value of social frailty on adverse outcomes in older adults living in the community. J Am Med Dir Assoc.

[77] Garre-Olmo J., Calvó-Perxas L., López-Pousa S., de Gracia, Blanco M., Vilalta-Franch J. (2013). Prevalence of frailty phenotypes and risk of mortality in a community-dwelling elderly cohort. Age Ageing.

[78] Lian Y., Yang L., Gao M., Jia C.-X. (2021). Relationship of frailty markers and socioeconomic status to incidence of depressive symptoms in a community cohort. J Am Med Dir Assoc.

[79] Teo N., Gao Q., Nyunt M.S.Z., Wee S.L., Ng T.-P. (2017). Social frailty and functional disability: findings from the Singapore longitudinal ageing studies. J Am Med Dir Assoc.

[80] Pek K., Chew J., Lim J.P., Yew S., Tan C.N., Yeo A. (2020). Social frailty is independently associated with mood, nutrition, physical performance, and physical activity: insights from a theory-guided approach. Int J Env Res Public Health.

[81] Yamada M., Arai H. (2018). Social frailty predicts incident disability and mortality among community-dwelling Japanese older adults. J Am Med Dir Assoc.

[82] Armstrong J.J., Andrew M.K., Mitnitski A., Launer L.J., White L.R., Rockwood K. (2015). Social vulnerability and survival across levels of frailty in the Honolulu-Asia aging study. Age Ageing.

[83] Gobbens R.J.J., van Assen M.A.L.M., Luijkx K.G., Wijnen-Sponselee M.T., Schols J.M.G.A. (2010). The Tilburg frailty indicator: psychometric properties. J Am Med Dir Assoc.

